# Preclinical Evaluation of hnRNPA2B1 Antibody in Human Triple-Negative Breast Cancer MDA-MB-231 Cells via PET Imaging

**DOI:** 10.3390/pharmaceutics14081677

**Published:** 2022-08-12

**Authors:** Abhinav Bhise, Hyun Park, Woonghee Lee, Swarbhanu Sarkar, Yeong Su Ha, Subramani Rajkumar, Bora Nam, Jeong Eun Lim, Phuong Tu Huynh, Kiwoong Lee, Ji-Yoon Son, Jung Young Kim, Kyo Chul Lee, Jeongsoo Yoo

**Affiliations:** 1 Department of Molecular Medicine, BK21 Plus KNU Biomedical Convergence Program, School of Medicine, Kyungpook National University, Daegu 41944, Korea; 2 Division of Applied RI, Korea Institute of Radiological and Medical Science, Seoul 01812, Korea

**Keywords:** hnRNPA2B1, immuno-PET, pretargeting, triple-negative breast cancer, nuclear imaging

## Abstract

Triple-negative breast cancer (TNBC) does not express estrogen receptor, progesterone receptor, and human epidermal growth factor receptor 2. Because TNBC lacks the expression of commonly targeted receptors, it is challenging to develop a new imaging agent for this cancer subtype. Heterogeneous nuclear ribonucleoproteins (hnRNPs) are RNA–protein complexes that have been linked to tumor development and progression. Considering the high expression of hnRNPA2B1, an hnRNP subtype, in TNBC MDA-MB-231 cells, this study aimed to develop a novel hnRNPA2B1 antibody-based nuclear imaging agent. The hnRNPA2B1-specific antibody was radiolabeled with ^64^Cu and evaluated in vitro and in vivo. The trans-cyclooctene (TCO) was functionalized on the antibody to obtain hnRNP-PEG_4_-TCO and reactive tetrazine (Tz) on the ultrastable bifunctional chelator PCB-TE2A-alkyne to yield PCB-TE2A-Tz for the inverse electron demand Diels–Alder reaction. The ^64^Cu-radiolabeled antibody was administered and imaged at 1–18 h time points for conventional imaging. Alternatively, the unlabeled antibody conjugate was administered, and 48 h later radiolabeled ^64^Cu-PCB-TE2A-Tz was administered to the same mice for the pretargeting strategy and imaged at the same time intervals for direct comparison. The tumor was successfully visualized in both strategies, and comparatively, pretargeting showed superior results. The ^64^Cu-PCB-TE2A-Tz was successfully clicked at the tumor site with hnRNP-PEG_4_-TCO and the non-clicked were concurrently eliminated. This led to increase the tumor uptake with extremely high tumor-to-background ratio manifested by positron emission tomography (PET) imaging and biodistribution studies.

## 1. Introduction

Triple-negative breast cancer (TNBC) is an aggressive subtype of breast cancer that does not express estrogen receptor (ER), progesterone receptor (PR), and human epidermal growth factor receptor 2 (HER2) on the cell surface [1,2]. Although TNBC accounts for only 15–20% of new breast cancer diagnoses, it is responsible for the majority of breast-cancer-related deaths owing to the lack of targeted therapy [3,4]. Recurrence and metastasis after chemotherapy and radiotherapy are the primary causes of mortality [5,6]. Owing to the lack of targetable receptors, it is also difficult to develop an imaging agent that targets TNBC. Although ^18^F-fluorodeoxyglucose positron emission tomography ([^18^F]FDG-PET) is the current gold standard for breast PET imaging, it has disadvantages such as non-specific internalization as well as low specificity and sensitivity, particularly for tumors < 1 cm^3^ [7,8].

Heterogeneous nuclear ribonucleoproteins (hnRNPs) constitute a family of RNA–protein complexes found in the nucleus of cells during gene transcription and subsequent posttranscriptional modification of pre-mRNA [9]. This family includes about 20 major polypeptides, including hnRNPs A1–U [10]. HnRNPs are involved in the regulation of mRNA stability and translation in several cancer types. Altered mRNA metabolism in several cancers supports the higher expression of hnRNPs in tumor tissues than in other tissues. HnRNPs play various roles in tumor apoptosis, angiogenesis, cell invasion, and metastasis [9,11]. Particularly, hnRNPA2B1, an hnRNP subtype, is an essential factor in tumor development and progression [12,13,14,15]. HnRNPA2/B1 has been identified as a new prognostic biomarker for breast cancer [16]. All these findings imply that hnRNPA2B1 could be a novel tumor target and a biomarker for monitoring treatment response and evaluating prognosis; however, to the best of our knowledge, no imaging agents based on hnRNPA2B1 antibody have been reported to date. High expression of hnRNPA2B1 in human TNBC MDA-MB-231 cells has been reported in several studies [17,18,19], hence, we considered the development of an hnRNPA2B1 antibody-based nuclear imaging agent for TNBC MDA-MB-231 cells.

**Figure 1:**
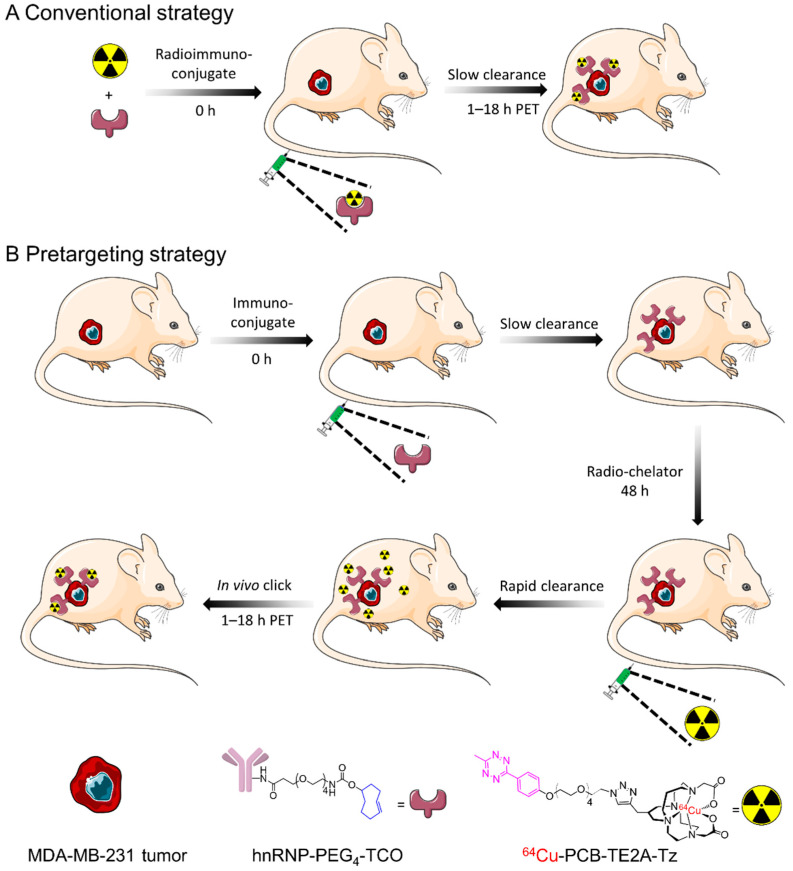
Schematic illustration depicting the (**A**) conventional strategy and (**B**) steps involved in pretargeting strategy using tetrazine (Tz)-trans-cyclooctene (TCO) reactive groups for bioorthogonal click reaction.

Immuno-positron emission tomography (immuno-PET) is a non-invasive and highly sensitive nuclear imaging technique where tumor-specific mAbs are radiolabeled with positron-emitting isotopes [20]. Immuno-PET enables visual monitoring of the pharmacokinetics of antibodies in vivo, thereby provides crucial information, such as the location of tumors. Typically, immuno-PET can be achieved through two strategies: “conventional” or “pretargeting” (Figure 1) [21]. Conventionally, mAbs have been radiolabeled directly with radioisotopes and studied preclinically/clinically for decades [22,23]. However, there are a few shortcomings associated with the conventional strategy, because of the long biological half-life of mAbs requiring days to circulate, increased radiation-burden on non-targeted tissues, and lower tumor-to-background ratio [24]. The pretargeting strategy overcomes the above problems associated with the conventional strategy [25]. Pretargeting is a multistep strategy, where the targeting moiety is separated from radiation source, and to date different approaches have already been employed and discussed [25,26]. Owing to the advantages of tumor-specific mAbs and the fastest bioorthogonal reaction (10^6^ M^−1^s^−1^) between tetrazine (Tz) and trans–cyclooctene (TCO), it was preferred in this study [27,28]. Additionally, pretargeting allows the use of mid-lived radionuclide such as ^64^Cu (*t*_1/2_12.7 h). In pretargeting, ^64^Cu radio-metal has been utilized in the development of tumor diagnosis [29,30,31]. Generally, bifunctional chelator (BFC) is required to attach radio-metal to mAbs. These BFCs are attached to the available amino acids, such as lysine residues, on antibodies [32]. In vivo stability of BFCs is a crucial factor in radiometal-based diagnosis [33]. Demetallation can cause deleterious effects on the non-targeted tissues as well as alter the information in diagnosis [34,35]. So far, a wide range of BFCs has been introduced to the antibodies, including common acyclic chelators [30,36,37] and polyazamacrocyclic chelators [38,39,40,41,42]. In this study, we utilized the recently reported by our group PCB-TE2A-Tz BFC for better in vivo performance and bioorthogonal click reaction [43].

Herein, we utilized the hnRNPA2B1 mAb for nuclear imaging of TNBC MDA-MB-231 tumor models. The hnRNPA2B1 mAb was conjugated with the TCO-PEG_4_-NHS ester to obtain hnRNP-PEG_4_-TCO conjugate. Further, it was clicked with the ^64^Cu-PCB-TE2A-Tz to yield the radioimmunoconjugate ^64^Cu-PCB-hnRNP. As a proof of concept, a comparative evaluation was undertaken between conventional and pretargeting strategies. The ^64^Cu-PCB-hnRNP was intravenously administered to TNBC tumor mice for conventional immuno-PET; for pretargeting, ^64^Cu-PCB-TE2A-Tz was administered 48 h after administrating the non-radiolabeled hnRNP-PEG_4_-TCO conjugate. Both strategies showed good tumor uptake. In pretargeting, the fast clearance of the radiochelator from the mice led to a lower radiation burden on the healthy tissues. Consequently, high tumor uptake with high tumor-to-background ratios were obtained than those obtained using conventional strategy at earlier time points.

## 2. Materials and Methods

### 2.1. General Information

All required reagents and solvents were purchased from Sigma–Aldrich (St. Louis, MO, USA) except where stated otherwise. The clickable PCB-TE2A-alkyne chelator was purchased from Future-Chem (Seoul, Korea) and other clickable linkers, such as methyl-T_Z_-PEG_4_-N_3_ and TCO-PEG_4_-NHS ester, were purchased from Click Chemistry Tools (Scottsdale, AZ, USA). The reactions involving tetrazine (Tz) moieties were carried out by covering the reagents with aluminum foil to protect them from light. The synthesized molecule was purified via high-performance liquid chromatography (HPLC) with >99% purity and then used in this study. The hnRNPA2/B1 (B-7) mAb was purchased from SantaCruz (Dallas, TX, USA). The antibody conjugate was freshly prepared every time before use in the experiments. All aqueous solutions required in this study were prepared by using deionized water (>18.2 mΩ cm^−1^) obtained from the Milli-Q water purification system (Millipore, Burlington, MA, USA). The MC50 cyclotron (Scanditronix, Vislanda, Sweden) at KIRAMS (Seoul, Korea) was used to produce ^64^Cu via the nuclear reaction of ^64^Ni(p, n)^64^Cu.

### 2.2. Radiosynthesis of ^64^Cu-PCB-TE2A-Tz

^64^Cu-labeled PCB-TE2A-Tz for pretargeting and other experiments was prepared as previously described [43]. Briefly, PCB-TE2A-Tz (10 μg in DMSO) was added to a mixture of ^64^CuCl_2_ (370–444 MBq, 10 μL, 0.01 M HCl) in 100 μL of 0.1 M NH_4_OAc buffer (pH 8.2). The resulting reaction mixture was incubated at 95 °C for 1 h. The radiochemical yield was assessed via radio-thin layer chromatography (radio-TLC) using C18 TLC plates (Merck) developed in 1:1 CH_3_CN:H_2_O (retention factor; R_f_ = 0.24). The R_f_ is the ratio between the solute distance travelled and the solvent distance travelled on TLC plates. The labeled ^64^Cu-PCB-TE2A-Tz was purified via HPLC (Waters Corp., Milford, MA, USA) and Bioscan Flow-Count detector (Berlin, Germany) using a Waters X-Bridge reversed-phase C18 column (5 μm, 4.6 × 150 mm). It was eluted at the flowrate of 1 mL/min with a mobile phase consisting of 0.1% trifluoroacetic acid (TFA) both in water (solvent A) and acetonitrile (solvent B). The gradient program was set as follows: 0% to 20% B for 5 min; 20% to 90% B from 5 to 25 min; and 90% to 0% B from 25 to 40 min. The ^64^Cu-PCB-TE2A-Tz was eluted at a retention time of 25 min under these gradient conditions. The fractions were collected as 200 µL per test tube and were evaporated to dryness on a rotary evaporator and collected in phosphate-buffered saline (PBS; 100 µL, pH 7.4) for further biological and non-biological experiments. The non-decay corrected isolated yield of the chelate was approximately 60%. The radiochemical purity of the isolated ^64^Cu-PCB-TE2A-Tz was analyzed using an Agilent infinity 1260 HPLC system (Agilent, CA, USA) and a Berthhold Technologies Lumo detector (Bad Wildbad, Germany) equipped with a Waters X-Bridge C18 column (5 μm, 4.6 × 150 mm) eluted at a flowrate of 1 mL/min with a gradient consisting of 0.1% TFA both in water (solvent A) and acetonitrile (solvent B). The gradient program was set as 20% B for 15 min; 20% to 90% B from 15 to 30 min; and 90% to 0% B from 30 to 40 min.

### 2.3. In Vivo Stability of ^64^Cu-PCB-TE2A-Tz

A solution of ^64^Cu-PCB-TE2A-Tz (approximately 11.1 MBq in 200 μL PBS, pH 7.4) was injected intravenously into healthy BALB/c mice. The mice were 6 weeks old and weighed around 19−20 g each (*n* = 3). The animals were euthanized 30 min after injection, and the blood, liver, and kidneys were collected in a tube and snap-frozen after measuring the radioactivity on a dose calibrator. To determine the stability of the radio-labeled chelator in blood, blood samples (0.3–0.5 mL) were centrifuged at 13,000 rpm at 4 °C, and around 400 μL of plasma was collected and ACN:H_2_O:TFA (100 µL, 50:45:5) was added. This was then centrifuged for 10 min at 13,000 rpm at 4 °C. The clear supernatant was filtered through a 0.22 μm filter, and the filtrate was injected into a reverse-phase radio HPLC X-Bridge RP C18 column (5 μm, 4.6 × 150 mm); it was eluted with a mobile phase consisting of 0.1% TFA/H_2_O (solvent A) and 0.1% TFA/CH_3_CN (solvent B), and a gradient consisting of 1% B to 70% B in 20 min at a flow rate of 1 mL/min. The radioactivity in each collected fraction (1.0 mL/tube) was then measured using a γ counter (Wallach, Turku, Finland). To determine the stability of ^64^Cu-PCB-TE2A-Tz in the liver and kidneys, the harvested organs were homogenized under ice-cold conditions. Each sample was suspended in PBS (1 mL) and vigorously vortexed for 5 min. The homogenates were then centrifuged for 10 min at 13,000 rpm to separate the solution from the bulk protein. To each supernatant (200 μL), ACN:H_2_O:TFA (100 µL, 50:45:5) was added, and the solution was centrifuged for 10 min at 13,000 rpm at 4 °C. The supernatant was then filtered through a 0.22 μm filter, and the filtrate was injected into a reverse-phase HPLC system the same as described for the blood. The radioactivity in each collected fraction (1.0 mL/tube) was then measured using a γ counter (Wallach, Turku, Finland). The percent demetallation was calculated as a ratio of counts of demetallated ^64^Cu and the counts of intact ^64^Cu-PCB-TE2A-Tz. Due to a time-limited schedule, two different HPLC instruments were used for sample analysis, resulting in two different retention times for the peaks, 25 and 30 min.

### 2.4. Conjugation of TCO-PEG_4_-NHS Ester to hnRNPA2B1 Antibody

**Scheme 1:**
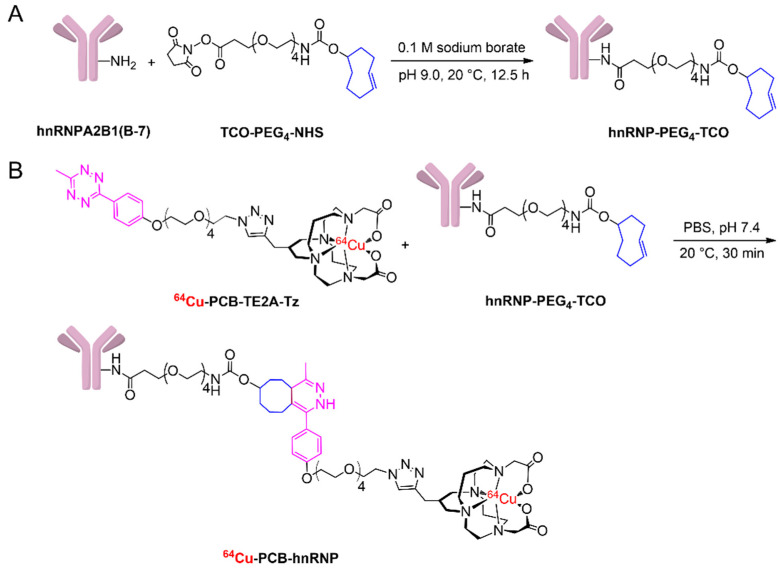
Preparation of hnRNPA2B1 antibody conjugate with TCO-PEG_4_-NHS ester (**A**); ex vivo click reaction between ^64^Cu-radiolabeled PCB-TE2A-Tz and the TCO-conjugated antibody (**B**).

To a solution of hnRNPA2B1 (200 µg in 1 mL, 0.0052 μmol, 1 eq.) and 0.1 M sodium tetraborate buffer (pH 9.0, 100 μL), TCO-PEG_4_-NHS ester (27 μg, 0.052 μmol, 10 eq.) was added and the resulting reaction vial was moderately thermomixed at 20 °C for 12.5 h. Then, the crude antibody conjugate was purified using a Centricon YM-3 kDa centrifugal filter unit (Millipore, Burlington, MA, USA) via centrifugation at 12,000 rpm for 7 min and washed with PBS three times to remove the unreacted TCO-PEG_4_-NHS ester. The concentrated antibody conjugate collected in 250 μL of PBS was considered 100% yield. The purified hnRNP-PEG_4_-TCO (200 µg) conjugate was aliquoted accordingly and stored at 4 °C prior to use (Scheme 1A).

### 2.5. Preparation of the Radioimmunoconjugate

The solution of hnRNP-PEG_4_-TCO in 50 μL of PBS (pH 7.4) was added to the vial containing purified ^64^Cu-PCB-TE2A-Tz (approximately 25.9 MBq in 50 μL of PBS; pH 7.4), and the resulting reaction mixture was incubated at 20 °C for 30 min on a thermomixer (Scheme 1B). The ex vivo labeling of antibody conjugate was confirmed via instant radio-TLC on a silica gel medium developed in 25% 0.1 M NH_4_OAc:MeOH. The purified radiolabeled antibody was then isolated using centrifugal filter units with a 10 kDa molecular weight cutoff (Sartorius Vivacon), washed three times, and collected in PBS, and its radiochemical purity was tested using the above-mentioned radio-TLC specifications. The radiochemical purity was found to be >99%. The radioimmunoconjugate remained at baseline, and ^64^Cu-PCB-TE2A-Tz had an R_f_ value of 0.58 on instant radio-TLC plates developed in 25% 0.1 M NH_4_OAc:MeOH (Figure S1).

### 2.6. In Vitro Serum and In Vivo Stability Studies of the Radioimmunoconjugate

In vitro serum stability was assessed by incubating approximately 3.7 MBq of the radioimmunoconjugate ^64^Cu-PCB-hnRNP in PBS or fetal bovine serum (FBS) at 37 °C. Samples were withdrawn using a micropipette at 0, 1, 2, 4, 8, 16, 24, 48, and 72 h post-addition of PBS or FBS and spotted on iTLC-SG to run in 25% 0.1 M NH_4_OAc:MeOH. The in vitro stability studies were performed in triplicate. For in vivo stability, approximately 11.1 MBq of ^64^Cu-PCB-hnRNP was administered through intravenous injection to 8-week-old BALB/c mice (*n* = 3). After 60 min post injection (p.i.), mice were euthanized to harvest the blood, liver, and kidneys and placed in an ice bath. The in vivo stability was determined by directly loading approximately 100 µL of blood on a pre-activated PD10 column and eluting with 20 mL of normal saline. The radioactivity in the collected fractions was measured using a γ counter (Wallach, Turku, Finland). To determine the stability in the liver and kidneys the organs were homogenized in ice-cold conditions with addition of PBS (1 mL). Further, the homogenized organs were vortexed and centrifuged at 13,000 rpm for 10 min at 4 °C. The supernatant (100 µL) was loaded on the pre-activated PD10 column and the same protocol was followed as described for the blood. The PD10 column was activated with 2% BSA and washed with normal saline before loading the samples. The control experiments were carried out by loading approximately 1.85 MBq of ^64^Cu-PCB-hnRNP on the PD10 column to determine the elution time of the intact radioimmunoconjugate.

### 2.7. Western Blotting for hnRNPA2B1 Expression

Proteins were extracted from MDA-MB-231, U87MG, MCF-7, and HepG2 cells by using 1× RIPA buffer (ThermoFisher Scientific, Waltham, MA, USA). The amount of protein from each cell was 20 μg. Protein samples were separated on 10% SDS-PAGE gels and then transferred to polyvinylidene fluoride (PVDF) membranes (GE Healthcare, Wien, Austria). The membrane was blocked with 5% skim milk (MB cell, Seoul, Korea) for 4 h. Western blotting was performed using antibodies against hnRNPA2/B1 (Santa Cruz Biotechnology, Inc., Santa Cruz, CA, USA) and β-actin (Santa Cruz Biotechnology). For horseradish peroxidase-conjugated secondary antibody, anti-mouse (GeneTex, Irvine, CA, USA) was detected using the ECL solution (BioRad, Hercules, CA, USA).

### 2.8. Cell Uptake Studies

Cells (1 × 10^5^/well) were incubated at 37 °C under, 5% CO_2_ conditions for 24 h prior to the start of the experiment. The radioimmunoconjugate uptake was measured by incubating approximately 0.037 MBq of the compound with four different cells lines (MDA-MB-231, U87MG, MCF-7, and HepG2) in 6-well plates for 60 min. The cells were then gently washed with PBS (0.5 mL × 2) and detached via treatment with trypsin-EDTA (0.5 mL). The radioactivity in the cells was measured using a γ counter (Wallach, Turku, Finland). The uptake was expressed as a proportion of the injected dose.

### 2.9. Animal Models

All animal experiments were performed in agreement with the approval authority—the Animal Care and Use Committee of Kyungpook National University (approval no. 2019-0101). The biodistribution studies and PET/computed tomography (CT) imaging models were prepared in female BALB/c nu/nu nude mice (6-weeks-old). Briefly, for the conventional strategy, 5 × 10^6^ and 1 × 10^7^ MDA-MB-231 cells were inoculated (right flank and on the left-side mammary fat pad), whereas for pretargeting cells were inoculated only on the right flank. For PET/CT studies, approximately 11.1 MBq, and for biodistribution studies, approximately 0.74 MBq were injected. The different amounts were injected to avoid saturation of counts on the highly sensitive γ counter.

### 2.10. Biodistribution Studies

Biodistribution studies of ^64^Cu-PCB-TE2A-Tz and its radioimmunoconjugate were performed in 6-week-old MDA-MB-231 tumor-bearing female BALB/c nude mice. For pretargeting studies, ^64^Cu-PCB-TE2A-Tz (approximately 0.74 MBq in 200 μL of PBS per mouse) was injected after 24 or 48 h of hnRNP-PEG_4_-TCO (40 μg) administration through the tail veins of the anesthetized mice (*n* = 3). The radioimmunoconjugate ^64^Cu-PCB-hnRNP was also injected at approximately 0.74 MBq in 200 μL of PBS (*n* = 3). Thereafter, all animals were euthanized after 1, 4, and 18 h since injection for radioimmunoconjugate and clearance studies. For pretargeting, the mice were euthanized at 4 and 8 h p.i. and the organs (heart, lungs, skin, fat, bones, spleen, kidneys, intestines, liver, gall bladder, and tumor tissues) were collected, weighed after blood was withdrawn, and subsequently analyzed using a γ counter (Wallach, Turku, Finland) for the respective studies. The proportion of the injected dose per gram (% ID/g) in each sample was calculated after comparison with a weighed and counted standard. Competitive blocking studies were performed to examine tumor-associated activity. For blocking, two separate syringes were prepared, one with the hnRNPA2B1 antibody (50 µg, 19.2-fold) and a second syringe with the radioimmunoconjugate ^64^Cu-PCB-hnRNP (approximately 0.74 MBq, 2.6 µg). In the MDA-MB-231 tumor mice (*n* = 2), first hnRNPA2B1 mAb was injected and immediately ^64^Cu-PCB-hnRNP was injected through the tail vein. At 4 h p.i. mice were euthanized and organs were harvested similarly as described for other biodistribution studies. The harvested tissues were weighed and analyzed using a γ counter (Wallach, Turku, Finland) to determine the accumulation of decay corrected radioactivity as %ID/g. The tumor-to-blood (T/B), tumor-to-muscle (T/M), tumor-to-kidney (T/K), and tumor-to-liver (T/L) values were also determined.

### 2.11. Micro PET/CT Imaging

Micro PET/CT images were obtained from an integrated nano-Scan PET/CT scanner (PET 82S, Mediso, Budapest, Hungary) for various time points: 1, 2, 4, and 18 h. The BALB/c nude mice bearing subcutaneous MDA-MB-231 tumor xenografts were pretargeted with hnRNP-PEG_4_-TCO (40 μg) 48 h before the injection of ^64^Cu-PCB-TE2A-Tz (approximately 11.1 MBq). The radioimmunoconjugate of ^64^Cu-PCB-hnRNP (approximately 11.1 MBq) was administered in mice bearing subcutaneous MDA-MB-231 xenografts and mammary fat pad orthotopic models. For both strategies, administration was completed via the tail veins following anesthesia with 2% isoflurane in O_2_ (Hana Pharm Co., Ltd., Seoul, Korea). The mice were anesthetized, placed in the prone position, and images were collected. PET and CT scanning were performed for 20 and 10 min, respectively, at early time points (1, 2, and 4 h), whereas for 18 h p. i., they were scanned for 45 and 10 min, respectively. Scans were reconstructed using the Mediso Tera-Tomo three-dimensional iterative algorithm with corrections for the interaction depth, radionuclide decay, detector normalization, crystal dead time, and attenuation. Reconstruction was performed using the following settings: 1:3 coincidence mode, four iterations, three subsets, normal regularization, spike filter on, 0.4 mm voxel size, and 400–600 keV energy window. The PET and CT images were co-registered automatically. Analysis of the acquired PET/CT images was performed using the Mediso Inter View Fusion software package. The PET quantification was expressed as standardized uptake values (SUV).

## 3. Results

### 3.1. Synthesis and Stability of ^64^Cu-PCB-TE2A-Tz

The propylene cross-bridge chelator was synthesized, characterized, and radiolabeled as previously reported [43]. The high in vivo stability of radiolabeled chelator is essential for biological studies, as demetallation can cause an unnecessary radiation burden on healthy tissues as well as result in a low tumor-to-background ratio. The in vivo stability studies were performed in healthy BALB/c mice. There was no considerable indication of demetallation in the blood, liver tissues, and kidney tissues. The radiolabeled chelator showed good stability in the blood (87.62 ± 1.40%) and liver (82.20 ± 4.28%) but its stability was slightly reduced in the kidneys (71.69% ± 8.23%) (Figures S2 and S3).

### 3.2. Western Blot and Cellular Uptake Studies

**Figure 2:**
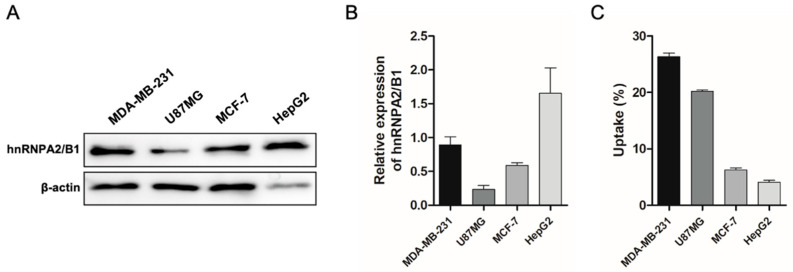
Representative Western blot analysis showing hnRNPA2B1 expression levels in four cancer cell lines using hnRNPA2B1-specific monoclonal antibody. β-actin was used as a loading control (**A**). Band densities were measured using ImageJ software (*n* = 4) (**B**). Cellular uptake studies of the radioimmunoconjugate ^64^Cu-PCB-hnRNP in different cancer cell lines (**C**).

Western blotting was performed on normalized protein concentration of cell lysates. In this study, the U87MG (glioma), MCF-7 (breast cancer), HepG2 (human liver cancer), and MDA-MB-231 (TNBC) cells were studied to determine the expression level of hnRNPA2B1. The concentration of the loading “housekeeping” protein (β-actin; ~42 kDa) in all cells appeared to be comparable. The target cancer cell MDA-MB-231 showed strong expression of hnRNPA2B1 at 36/38 kDa (Figure 2A,B). These results validate the potential of hnRNPA2B1 antibody for TNBC imaging.

Subsequent, cellular uptake studies were conducted in tumor cells as described above. After treating the radioimmunoconjugate ^64^Cu-PCB-hnRNP in the cellular suspension, the highest uptake was found for MDA-MB-231 cells (26.3 ± 1.1%) within just 1 h of incubation, and the lowest uptake (4.1 ± 0.6%) was found for HepG2 cells. The order of radioimmunoconjugate uptake in the tumor cell lines was MDA-MB-231 > U87MG > MCF-7 > HepG2 (Figure 2C). These in vitro results clearly demonstrated that hnRNPA2B1 antibody could bind to the TNBC MDA-MB-231 cells. Moreover, the hnRNPA2B1 expression level varied in different cancer types, resulting in these divergent findings in cellular uptake studies [17,18]. Despite unlikely results obtained from Western blot and cellular uptake studies for other cell lines, the target MDA-MB-231 showed consistent results in both experiments. Ultimately, after confirming the in vitro results we initiated our animal experiments with TNBC models.

### 3.3. Clearance of the Radiochelator ^64^Cu-PCB-TE2A-Tz

To monitor ^64^Cu-PCB-TE2A-Tz behavior in vivo and elimination profile via hepatically or renally, approximately 1.85 MBq of the radiochelator was administered through intravenous injection in MDA-MB-231 tumor xenograft mice in the absence of antibody. At each of the 1, 4, and 18 h time point, respective mice were euthanized to quantify the amount of radioactivity remaining in the organs using a γ counter. At 1 h, the radiolabeled chelator was cleared from the bloodstream, leaving only 0.62 ± 0.25% ID/g in the lungs, 0.43 ± 0.15% ID/g in the kidneys, 16.02 ± 0.35% ID/g in the intestines, 0.67 ± 0.05% ID/g in the liver, and 7.08 ± 0.34% ID/g in the gallbladder, implying hepatic clearance. The results obtained from biodistribution studies revealed that within 1 h p.i., the radiolabeled ^64^Cu-PCB-TE2A-Tz was rapidly cleared from the mice showing no non-specific affinity toward the tumor (Figure S7). Subsequently, the biodistribution results were corroborated with PET imaging (Figure S8). The PET data further confirmed that the radiochelator was cleared rapidly with increasing time. However, the PET and biodistribution studies showed distinguishable uptake of the chelator in the gallbladder (Figure S13). This dual rapid hepatic as well as renal clearance benefites the overcoming of the unwanted burden of radiation.

### 3.4. In Vitro and In Vivo Stability of ^64^Cu-PCB-hnRNP

**Figure 3:**
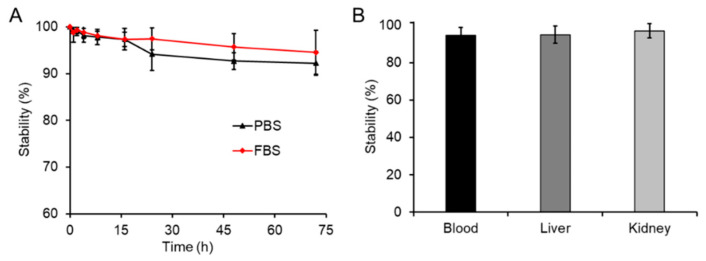
In vitro stability studies of the radioimmunoconjugate ^64^Cu-PCB-hnRNP in PBS and FBS (*n* = 3) at various time points (**A**). In vivo stability studies of radioimmunoconjugate ^64^Cu-PCB-hnRNP in the blood, liver, and kidneys of BALB/c mice (*n* = 3) 1 h p.i. (**B**).

Initially, we monitored the in vitro stability of ^64^Cu-PCB-hnRNP over 3 days of incubation in serum at 37 °C. The ^64^Cu-PCB-hnRNP showed no considerable degradation either in FBS or in PBS. Overall >92% in PBS and >94% in FBS of the ^64^Cu-radiolabeled antibody was intact for up to 72 h of incubation (Figure 3A and Figure S5). Afterwards, the in vivo stability was determined by the collection of the blood, liver, and kidneys after 1 h p.i., and the proportion of intact radioimmunoconjugate in each sample was calculated. The counts of intact ^64^Cu-PCB-hnRNP were separated from the demetallated metabolites by PD10 size exclusion chromatography (Figure 3B and Figure S6). The obtained results indicate that the radiochelator is more stable when conjugated with the antibody compared to the radiochelator itself (Figure S2). The radioimmunoconjugate showed no substantial differences in the blood, liver, or kidneys, and it was stable up to 94.67 ± 4.2%, 95.04 ± 4.6%, and 97.10 ± 3.85%, respectively. Overall, the ^64^Cu-PCB-hnRNP showed excellent stability in both serum and in vivo, which is indispensable for immuno-PET.

### 3.5. In Vivo Analysis of the Radioimmunoconjugate ^64^Cu-PCB-hnRNP

**Figure 4:**
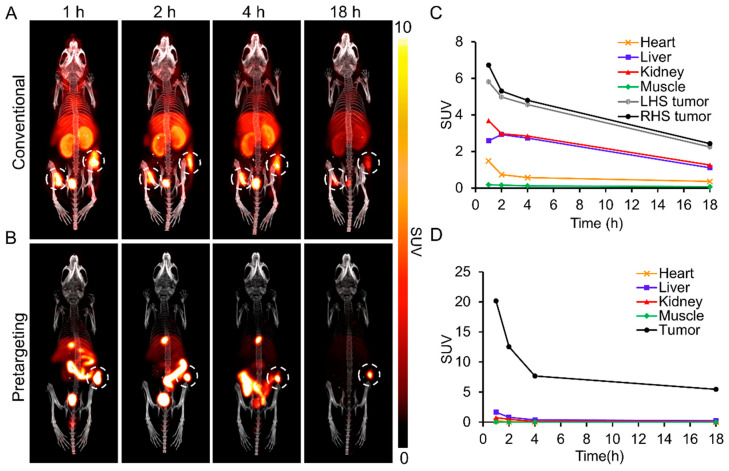
Maximum intensity projection PET/CT images of conventional (**A**) and pretargeting strategies (**B**) in female nude mice bearing MDA-MB-231 tumor xenografts. PET-quantified standardized uptake values of conventional and pretargeting strategies (**C**,**D**), respectively (*n* = 1 each). ^64^Cu-PCB-TE2A-Tz was administered 48 h after administration of hnRNP-PEG_4_-TCO for the pretargeting strategy. Xenografts are highlighted by a white dashed circle.

After the clearance and stability studies, the radioimmunoconjugate ^64^Cu-PCB-hnRNP was administered in an MDA-MB-231 tumor mouse. As the MDA-MB-231 tumor cells are highly metastatic, we intended to undertake this study in metastatic tumor models. For this, two tumor xenografts were grown in nude mice: one at the right-hand side flank (RHS) and another in the mammary fat pad on the left-hand side (LHS) of the same mice, but we were unable to generate metastatic tumor models. Nevertheless, at the 1 h time point, the radioimmunoconjugate successfully accumulated at the tumor, showing a strong signal in PET/CT imaging studies (Figure 4A and Figure S11). The PET quantification revealed the tumor standardized uptake value (SUV) of 6.7 and 2.4, and 5.8 and 2.1 at 1 and 18 h p.i. for the flank tumor and mammary fat pad tumor, respectively. The PET-quantified tumor-to-background ratio for both the LHS and RHS tumor was obtained as tumor-to-muscle (T/M) = 32.8, tumor-to-kidney (T/K) = 1.6, and tumor-to-liver (T/L) = 2.3 at 1 h p.i., and T/M = 30.7, T/K = 1.8, and T/L = 2.0 at 18 h p.i. for the LHS tumor. For the RHS tumor, similar tumor-to-background ratios were obtained for 1 and 18 h p.i.: T/M = 37.9, T/K = 1.8, and T/L = 2.6 at 1 h p.i.; T/M = 33.3, T/K = 1.9, and T/L = 2.2 at 18 h p.i. (Figure 4C).

**Figure 5:**
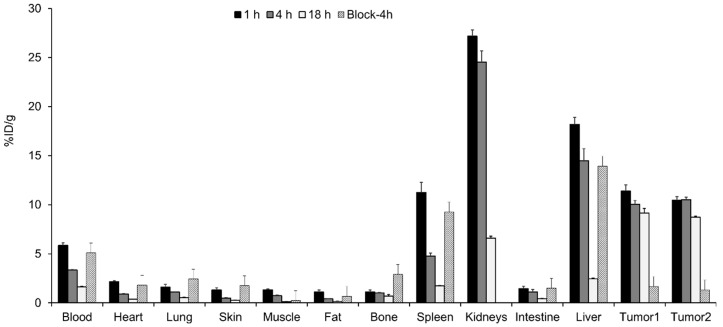
Biodistribution studies of the radioimmunoconjugate ^64^Cu-PCB-hnRNP in female nude mice bearing TNBC MDA-MB-231 tumors at different timepoints (Tumor 1 = left-hand side) and (Tumor 2 = right-hand side). An excess blocking dose of hnRNPA2B1 (50 μg; 19.2-fold) was given prior to administration of ^64^Cu-PCB-hnRNP and specificity was evaluated at the 4 h after injection.

In the biodistribution studies of the radioimmunoconjugates, the tumor uptake was 10.04 ± 0.37 and 9.12 ± 0.47% ID/g at 4 and 18 h, respectively, in the LHS tumor, and 10.51 ± 0.25 and 8.72 ± 0.09% ID/g at 4 and 18 h, respectively, in the RHS tumor. However, the PET and biodistribution studies revealed the slow renal and hepatic clearance of the radioimmunoconjugates. The blood, kidneys, liver, and spleen showed an uptake of 5.84 ± 0.28, 27.17 ± 0.65, 18.16 ± 0.73, and 11.24 ± 1.02% ID/g at 1 h, respectively, concurrently reducing at 18 h to 1.63 ± 0.06, 6.61 ± 0.01, 2.44 ± 0.08, and 1.75 ± 0.16% ID/g, respectively (Figure 5). The T/B, T/M, and T/L ratios of radioimmunoconjugate at 18 h p.i. were 5.5, 65.2, and 3.7, respectively for the LHS tumor, and 5.3, 62.3, and 3.5, respectively for the RHS tumor.

To further validate the hnRNPA2B1 specificity, blocking studies were performed (Figure 5). Prior to the administration of the ^64^Cu-PCB-hnRNP, the tumor was blocked by injecting excess hnRNPA2B1 mAb (19.2-fold) and at 4 h p.i. organs were harvested for the biodistribution studies. The obtained results revealed sufficient reduction of the tumor uptake of 1.68 ± 0.39%ID/g and 1.32 ± 0.11%ID/g for LHS and RHS tumor, respectively, which is only 17% and 13% of non-blocked uptakes. The activities in other organs were nearly similar in the blocking study. Moreover, these studies using radioimmunoconjugates suggested that the tumor can be clearly detected by the hnRNPA2B1 antibody.

However, along with the tumor, signals from non-targeted tissues were also evident. To overcome this, as a proof of concept, we employed a pretargeting strategy with the hnRNP-PEG_4_-TCO conjugate, which allows efficient circulation of the antibody and ultimately clears the non-bound antibodies from the bloodstream.

### 3.6. In Vivo Analysis Using the Pretargeting Strategy

**Figure 6:**
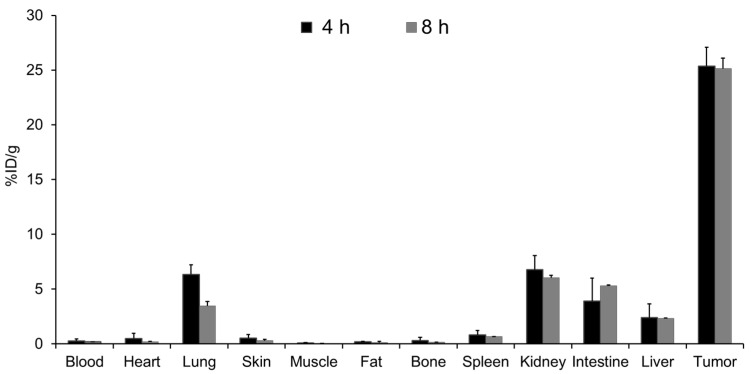
Biodistribution data of pretargeting strategy at 48 h post-injection of hnRNP conjugate in female nude mice bearing MDA-MB-231 tumor xenografts. Organs were harvested at 4 and 8 h after the administration of ^64^Cu-PCB-TE2A-Tz.

In the pretargeting studies, we first determined the optimal time required for the hnRNP-PEG_4_-TCO conjugate to circulate in the body by injecting antibody 24 and 48 h prior to injecting the radiochelator. The tumor uptake was observed 18.42 ± 42%ID/g at the 4 h time point when the antibody was injected 24 h prior (Figure S6). However, we conducted all other experiments by injecting hnRNP-PEG_4_-TCO 48 h before the radiolabeled ^64^Cu-PCB-TE2A-Tz, showing higher tumor uptake (Figure 6). The PET images showed that as time increased the signal from the tumor slowly decreased (Figure 4B,D and Figure S12). However, the signal from the abdominal region at 1 h p.i. was significantly reduced at 4 h, and at 18 h p.i. it was completely diminished. The rapid clearance from the background healthy tissues and organs was also an indication that the radiolabeled chelator was rapidly excreted. These PET images clearly showed that the pretargeting strategy can be achieved by the hnRNPA2B1 antibody to visualize the tumor at an earlier time point with less background interference. We also intended to apply this pretargeting strategy to visualize a tumor at a different location. The MDA-MB-231 xenografts were inoculated at the left shoulder on the female BALB/c nude mice. Even when the tumor location was changed from flank to shoulder, excellent tumor uptake was observed as shown in PET imaging (Figure S10). The PET quantification for pretargeting showed the highest tumor SUV of 20.2 at 1 h p.i. and 5.4 at 18 h p.i. The resulting tumor-to-organ ratios were found to be very high at 1 h p.i., T/M = 807.1, T/K = 27.0, and T/L = 12.3 and it peaked time-dependently to obtain T/M = 1111, T/K = 58.0, and T/L = 25.4 at 18 h p.i. (Figure 4D).

The ex vivo biodistribution was in good agreement with the obtained PET images (Figure 6). At 4 h p.i., the liver, kidneys, and tumor of the pretargeting mice showed high uptake of 2.14 ± 1.24, 6.78 ± 1.30, and 25.37 ± 1.70%ID/g, respectively, and this remained similar for up to 8 h for the liver (2.34 ± 0.04%ID/g), kidneys (6.03 ± 0.22%ID/g), and tumor (25.15 ± 0.95%ID/g). The resulting tumor-to-organ ratios were T/M = 314.1, T/B = 92.2, and T/L = 10.5 at the 4 h time point for pretargeting and were increased at the 8 h time point (T/M = 558.5, T/B = 127.5, and T/L = 10.8) (Table S1). In the pretargeting biodistribution studies, the later hour time points were not required as the radiolabeled chelator was cleared out rapidly with maximum tumor uptake and a high tumor-to-background ratio at 8 h p.i. In comparison, conventional tumor imaging showed lower tumor-to-organ ratios of T/M = 13.6, T/B = 2.9, and T/L = 0.6 at the 4 h time point, indicating that pretargeting reduced the background interference more efficiently (Table S1).

## 4. Discussion

Breast cancer remains the most frequently diagnosed female cancer and the second leading cause of cancer-related death among women [44]. TNBC, a subtype of breast cancer that lacks ER, PR, and HER2 expression, is associated with high metastasis and poor prognosis. The lack of targeted receptors in TNBC makes it difficult to develop targeted therapies and imaging agents for this cancer. To date, only few attempts to effectively diagnose TNBC have been reported. For instance, Henry et al. reported promising results in diagnosing MDA-MB-231 tumors by utilizing increased surface expression of transferrin receptor, a downstream event of MYC protein upregulation in TNBC, and compared the efficiency of ^18^F-FDG versus ^89^Zr-transferrin [7]. Cheng et al. tested the hypoxia imaging agent ^18^F-FMISO for the detection of hypoxia in the tumor microenvironment of TNBC [45]. Despite these attempts, the lack of definitive diagnostic tools for TNBC encourages us to investigate hnRNPA2B1 as a potential candidate for a promising biomarker. The hnRNPs comprise a family of RNA-binding proteins that contribute to nucleic acid metabolism, mRNA stabilization, and transcriptional and translational regulation. It is present at elevated levels in TNBC MDA-MB-231 (Figure 2A,B and Figure S4) [18,19]. The presence of hnRNPs and their role as a biomarker in breast cancer have been reported for decades [9,12]. However, for the first time, we explored hnRNPA2B1-specific antibody for immuno-PET imaging of TNBC MDA-MB-231 in mice. The antibody was functionalized with TCO (hnRNP-PEG_4_-TCO) and ^64^Cu-labeled BFC was functionalized with Tz (^64^Cu-PCB-TE2A-Tz) to visualize the tumor by PET imaging (Scheme 1).

By Western blotting, the hnRNPA2B1 expression levels were evaluated and found to be high in MDA-MB-231 (Figure 2A,B and Figure S4). The radiochelator and radioimmunoconjugate were found to be highly stable in vitro and in vivo (Figure 3 and Figure S2). The biodistribution studies demonstrated high tumor uptake in both tumors of 11.37 ± 0.65%ID/g and 10.44 ± 0.37%ID/g 1 h p.i. and slightly reduced tumor uptake of 9.12 ± 0.47%ID/g and 8.72 ± 0.09%ID/g at 18 h p.i., for left-hand side and right-hand side tumors, respectively (Figure 5). This high tumor accumulation of radioimmunoconjugate was in accordance with the highest (26.3 ± 1.1%) cellular uptake and Western blot analysis (Figure 2). Although the tumor uptake was high, the PET studies suggested that the directly radiolabeled radioimmunoconjugate needed more time for sufficiently high contrast between tumor and background (Figure 4A). Additionally, to evaluate specificity of ^64^Cu-PCB-hnRNP radioimmunoconjugate, subsequent blocking studies were performed. Blocking with unlabeled hnRNPA2B1 antibody effectively lowered the tumor uptake of ^64^Cu-PCB-hnRNP at 4 h by 1/6-fold and 1/8-fold for LHS and RHS tumors, respectively (Figure 5). Thus, the specificity of hnRNPA2B1 targeting TNBC MDA-MB-231 was confirmed by a blocking study.

A pretargeting strategy was applied to achieve higher tumor-to-background ratios at earlier time points. The initial biodistribution studies revealed a tumor uptake at 4 h p.i., of 18.42 ± 1.11%ID/g and 25.37 ± 1.69%ID/g for 24 and 48 h time points, respectively (Figure 6 and Figure S9). The 48 h pre-circulation of hnRNPA2B1 antibody increased tumor uptake by 25% compared to 24 h circulation. The tumor-to-muscle (T/M) ratio at 8 h p.i. was increased to exceptionally high 559 from 314 at 4 h p.i. in the 48 h pre-circulation of the hnRNPA2B1 conjugate. For comparison, in a ^64^Cu-based pretargeting studies conducted by Zeglis et al., the tumor uptake remained constant at around 4%ID/g from 1 to 24 h p.i. with a maximum T/M ratio of 27.0 ± 7.4 at 24 h [30]. Zeglis et al. also reported that when ^64^Cu-Tz-SarAr chelator was used in a pretargeting strategy, tumor uptake was 7.38 ± 2.02 at 24-h p.i. with a T/M ratio of 45.1 ± 8.6 [29].

The PET imaging also demonstrated the high tumor uptake of radioactivity mediated by hnRNPA2B1 within 1 h (Figure 4B). Tumor signals became evident over time while simultaneously reducing background noise. Overall, the PET and biodistribution studies of the pretargeting strategy highlights the successful preclinical evaluation of hnRNPA2B1 mAb for non-invasive and quantitative immuno-PET diagnosis of TNBC MDA-MB-231.

## 5. Conclusions

In conclusion, for the first time, TNBC MDA-MB-231 tumors were successfully detected with the hnRNPA2B1 mAb in animal models using high-sensitivity immuno-PET imaging. As a proof of concept, conventional and pretargeting approaches were employed and high tumor uptake was observed in both strategies. The tumor specificity of the hnRNPA2B1 antibody was successfully evaluated using a blocking study in the TNBC MDA-MB-231 tumor model. Compared to the conventional strategy using the ex vivo labeled hnRNPA2B1 antibody, the pretargeting strategy showed the higher tumor uptake with very minimal background noises. Finally, we believe that this study will provide insight into the further development of antibody-based TNBC imaging.

## Data Availability

Additional supporting data can be found in Supplementary Materials (S1-T1).

## Supplementary Materials

The following supporting information can be downloaded at: https://www.mdpi.com/article/10.3390/pharmaceutics14081677/s1, Figure S1: Radio-TLC of radioimmunoconjugate ^64^Cu-PCB-hnRNP; Figure S2: In vivo stability studies of ^64^Cu-PCB-TE2A-Tz; Figure S3: HPLC-chromatograms of in vivo stability of ^64^Cu-PCB-TE2A-Tz; Figure S4: Western blot analysis of hnRNPA2B1 expression levels; Figure S5: Radio-TLCs of in vitro stability studies of ^64^Cu-PCB-hnNRP; Figure S6: SEC-chromatograms of in vivo stability of ^64^Cu-PCB-hnNRP; Figure S7: Clearance biodistribution studies of ^64^Cu-PCB-TE2A-Tz in MDA-MB-231 tumor mice; Figure S8: Clearance MIP PET/CT images of ^64^Cu-PCB-TE2A-Tz in MDA-MB-231 tumor mice; Figure S9: Biodistribution studies of the 24 h pretargeting strategy; Figure S10: Pretargeted PET imaging of the MDA-MB-231 tumor at the left shoulder; Figure S11: The 2D sectional tumor imaging of the conventional strategy; Figure S12: The 2D sectional tumor imaging of the pretargeting strategy; Figure S13: Gallbladder 2D sectional tumor imaging of the pretargeting strategy; Table S1: Tumor-to-organ ratio by both conventional strategy and pretargeting strategy.

## References

[1] Aysola K., Desai A., Welch C., Xu J., Qin Y., Reddy V., Matthews R., Owens C., Okoli J., Beech D.J. (2013). Triple Negative Breast Cancer—An Overview. Hered. Genet..

[2] Kumar P., Aggarwal R. (2016). An overview of triple-negative breast cancer. Arch. Gynecol. Obstet..

[3] Yang R., Li Y., Wang H., Qin T., Yin X., Ma X. (2022). Therapeutic progress and challenges for triple negative breast cancer: Targeted therapy and immunotherapy. Mol. Biomed..

[4] Luo C., Wang P., He S., Zhu J., Shi Y., Wang J. (2022). Progress and Prospect of Immunotherapy for Triple-Negative Breast Cancer. Front. Oncol..

[5] Zhang C., Wang S., Israel H.P., Yan S.X., Horowitz D.P., Crockford S., Gidea-Addeo D., Clifford Chao K.S., Kalinsky K., Connolly E.P. (2015). Higher locoregional recurrence rate for triple-negative breast cancer following neoadjuvant chemotherapy, surgery and radiotherapy. Springerplus.

[6] Newton E.E., Mueller L.E., Treadwell S.M., Morris C.A., Machado H.L. (2022). Molecular Targets of Triple-Negative Breast Cancer: Where Do We Stand?. Cancers.

[7] Henry K.E., Dilling T.R., Abdel-Atti D., Edwards K.J., Evans M.J., Lewis J.S. (2018). Noninvasive ^89^Zr-Transferrin PET Shows Improved Tumor Targeting Compared with ^18^F-FDG PET in MYC-Overexpressing Human Triple-Negative Breast Cancer. J. Nucl. Med..

[8] Almuhaideb A., Papathanasiou N., Bomanji J. (2011). ^18^F-FDG PET/CT imaging in oncology. Ann. Saudi Med..

[9] Han N., Li W., Zhang M. (2013). The function of the RNA-binding protein hnRNP in cancer metastasis. J. Cancer Res. Ther..

[10] Krecic A.M., Swanson M.S. (1999). hnRNP complexes: Composition, structure, and function. Curr. Opin. Cell Biol..

[11] Carpenter B., MacKay C., Alnabulsi A., MacKay M., Telfer C., Melvin W.T., Murray G.I. (2006). The roles of heterogeneous nuclear ribonucleoproteins in tumour development and progression. Biochim. Biophys. Acta.

[12] Liu Y., Shi S.L. (2021). The roles of hnRNP A2/B1 in RNA biology and disease. Wiley Interdiscip. Rev. RNA.

[13] He Y., Smith R. (2009). Nuclear functions of heterogeneous nuclear ribonucleoproteins A/B. Cell Mol. Life Sci..

[14] Gu W., Liu W., Shen X., Shi Y., Wang L., Liu H. (2013). Emergence of heterogeneous nuclear ribonucleoprotein A2/B1 vs loss of E-cadherin: Their reciprocal immunoexpression profiles in human pancreatic cancer. Ann. Diagn. Pathol..

[15] He Y., Rothnagel J.A., Epis M.R., Leedman P.J., Smith R. (2009). Downstream targets of heterogeneous nuclear ribonucleoprotein A2 mediate cell proliferation. Mol. Carcinog..

[16] Ma Y., Yang L., Li R. (2020). HnRNPA2/B1 Is a Novel Prognostic Biomarker for Breast Cancer Patients. Genet. Test. Mol. Biomarkers.

[17] Li L., Wu M., Wang C., Yu Z., Wang H., Qi H., Xu X. (2018). beta-Asarone Inhibits Invasion and EMT in Human Glioma U251 Cells by Suppressing Splicing Factor HnRNP A2/B1. Molecules.

[18] Hu Y., Sun Z., Deng J., Hu B., Yan W., Wei H., Jiang J. (2017). Splicing factor hnRNPA2B1 contributes to tumorigenic potential of breast cancer cells through STAT3 and ERK1/2 signaling pathway. Tumour. Biol..

[19] Liu Y., Li H., Liu F., Gao L.B., Han R., Chen C., Ding X., Li S., Lu K., Yang L. (2020). Heterogeneous nuclear ribonucleoprotein A2/B1 is a negative regulator of human breast cancer metastasis by maintaining the balance of multiple genes and pathways. EBioMedicine.

[20] Van Dongen G.A., Visser G.W., Lub-de Hooge M.N., de Vries E.G., Perk L.R. (2007). Immuno-PET: A navigator in monoclonal antibody development and applications. Oncologist.

[21] Wei W., Rosenkrans Z.T., Liu J., Huang G., Luo Q.Y., Cai W. (2020). ImmunoPET: Concept, Design, and Applications. Chem. Rev..

[22] Liu S. (2008). Bifunctional coupling agents for radiolabeling of biomolecules and target-specific delivery of metallic radionuclides. Adv. Drug. Deliv. Rev..

[23] Peltek O.O., Muslimov A.R., Zyuzin M.V., Timin A.S. (2019). Current outlook on radionuclide delivery systems: From design consideration to translation into clinics. J. Nanobiotechnology.

[24] Knox S.J. (1995). Overview of studies on experimental radioimmunotherapy. Cancer Res..

[25] Liu G. (2018). A Revisit to the Pretargeting Concept-A Target Conversion. Front. Pharmacol..

[26] Patra M., Zarschler K., Pietzsch H.J., Stephan H., Gasser G. (2016). New insights into the pretargeting approach to image and treat tumours. Chem. Soc. Rev..

[27] Rondon A., Degoul F. (2020). Antibody Pretargeting Based on Bioorthogonal Click Chemistry for Cancer Imaging and Targeted Radionuclide Therapy. Bioconjugate Chem..

[28] Blackman M.L., Royzen M., Fox J.M. (2008). Tetrazine ligation: Fast bioconjugation based on inverse-electron-demand Diels-Alder reactivity. J. Am. Chem. Soc..

[29] Zeglis B.M., Brand C., Abdel-Atti D., Carnazza K.E., Cook B.E., Carlin S., Reiner T., Lewis J.S. (2015). Optimization of a Pretargeted Strategy for the PET Imaging of Colorectal Carcinoma via the Modulation of Radioligand Pharmacokinetics. Mol. Pharm..

[30] Zeglis B.M., Sevak K.K., Reiner T., Mohindra P., Carlin S.D., Zanzonico P., Weissleder R., Lewis J.S. (2013). A pretargeted PET imaging strategy based on bioorthogonal Diels-Alder click chemistry. J. Nucl. Med..

[31] Houghton J.L., Zeglis B.M., Abdel-Atti D., Sawada R., Scholz W.W., Lewis J.S. (2016). Pretargeted Immuno-PET of Pancreatic Cancer: Overcoming Circulating Antigen and Internalized Antibody to Reduce Radiation Doses. J. Nucl. Med..

[32] Kang M.S., Kong T.W.S., Khoo J.Y.X., Loh T.P. (2021). Recent developments in chemical conjugation strategies targeting native amino acids in proteins and their applications in antibody-drug conjugates. Chem. Sci..

[33] Wadas T.J., Wong E.H., Weisman G.R., Anderson C.J. (2010). Coordinating radiometals of copper, gallium, indium, yttrium, and zirconium for PET and SPECT imaging of disease. Chem. Rev..

[34] Boswell C.A., Sun X., Niu W., Weisman G.R., Wong E.H., Rheingold A.L., Anderson C.J. (2004). Comparative in vivo stability of copper-64-labeled cross-bridged and conventional tetraazamacrocyclic complexes. J. Med. Chem..

[35] Bass L.A., Wang M., Welch M.J., Anderson C.J. (2000). In vivo transchelation of copper-64 from TETA-octreotide to superoxide dismutase in rat liver. Bioconjugate Chem..

[36] Vosjan M.J., Perk L.R., Visser G.W., Budde M., Jurek P., Kiefer G.E., van Dongen G.A. (2010). Conjugation and radiolabeling of monoclonal antibodies with zirconium-89 for PET imaging using the bifunctional chelate p-isothiocyanatobenzyl-desferrioxamine. Nat. Protoc..

[37] Moi M.K., Meares C.F., McCall M.J., Cole W.C., DeNardo S.J. (1985). Copper chelates as probes of biological systems: Stable copper complexes with a macrocyclic bifunctional chelating agent. Anal. Biochem..

[38] Navarro A.S., Le Bihan T., Le Saec P., Bris N.L., Bailly C., Sai-Maurel C., Bourgeois M., Cherel M., Tripier R., Faivre-Chauvet A. (2019). TE1PA as Innovating Chelator for ^64^Cu Immuno-TEP Imaging: A Comparative in Vivo Study with DOTA/NOTA by Conjugation on 9E7.4 mAb in a Syngeneic Multiple Myeloma Model. Bioconjugate Chem..

[39] Yang H., Gao F., McNeil B., Zhang C., Yuan Z., Zeisler S., Kumlin J., Zeisler J., Benard F., Ramogida C. (2021). Synthesis of DOTA-pyridine chelates for ^64^Cu coordination and radiolabeling of alphaMSH peptide. EJNMMI Radiopharm. Chem..

[40] Farleigh M., Pham T.T., Yu Z., Kim J., Sunassee K., Firth G., Forte N., Chudasama V., Baker J.R., Long N.J. (2021). New Bifunctional Chelators Incorporating Dibromomaleimide Groups for Radiolabeling of Antibodies with Positron Emission Tomography Imaging Radioisotopes. Bioconjugate Chem..

[41] Boswell C.A., Regino C.A., Baidoo K.E., Wong K.J., Bumb A., Xu H., Milenic D.E., Kelley J.A., Lai C.C., Brechbiel M.W. (2008). Synthesis of a cross-bridged cyclam derivative for peptide conjugation and ^64^Cu radiolabeling. Bioconjugate Chem..

[42] Pandya D.N., Dale A.V., Kim J.Y., Lee H., Ha Y.S., An G.I., Yoo J. (2012). New macrobicyclic chelator for the development of ultrastable ^64^Cu-radiolabeled bioconjugate. Bioconjugate Chem..

[43] Lee W., Sarkar S., Pal R., Kim J.Y., Park H., Huynh P.T., Bhise A., Bobba K.N., Kim K.I., Ha Y.S. (2021). Successful Application of CuAAC Click Reaction in Constructing ^64^Cu-Labeled Antibody Conjugates for Immuno-PET Imaging. ACS Appl. Bio. Mater..

[44] Siegel R.L., Miller K.D., Jemal A. (2019). Cancer statistics, 2019. CA Cancer J. Clin..

[45] Cheng Z., Jiang X., Yang L., Shen T., Wang X., Lu H., Wu J., Xia C., Song B., Ai H. (2016). Micro PET imaging of ^18^F-Fluoromisonidazole in an MDA-MB-231 triple negative human breast cancer xenograft model. Transl. Cancer Res..

