# Supplementary material for: Preclinical Evaluation of hnRNPA2B1 Antibody in Human Triple-Negative Breast Cancer MDA-MB-231 Cells via PET Imaging
Source: Pharmaceutics. 2022 Aug 12;14(8):1677. doi: 10.3390/pharmaceutics14081677 (PMC9415040; doi:10.3390/pharmaceutics14081677)
Supplement: Supplementary file 1 [file pharmaceutics-14-01677-s001.zip › pharmaceutics-1727221-SI.pdf]

Supporting information

Preclinical Evaluation of hnRNPA2B1 Antibody in Human Triple-Negative Breast Cancer MDA-MB-231 Cells via PET Imaging

Table of contents:

S1. Radio-TLC of radioimmunoconjugate ^{64}Cu-PCB-hnNRP

S2. *In vivo* stability study of ^{64}Cu-PCB-TE2A-Tz

S3. HPLC-chromatograms of *in vivo* stability of ^{64}Cu-PCB-TE2A-Tz

S4. Western blot analysis of hnRNPA2B1 expression in various cells lines

S5. Radio-TLCs of *in vitro* stability studies of radioimmunoconjugate ^{64}Cu-PCB-hnNRP

S6. SEC-chromatograms of *in vivo* stability of radioimmunoconjugate ^{64}Cu-PCB-hnNRP

S7. Clearance biodistribution studies of the ^{64}Cu-PCB-TE2A-Tz in the MDA-MB-231 tumor mice

S8. Clearance PET/CT images of the ^{64}Cu-PCB-TE2A-Tz in the MDA-MB-231 tumor mice

S9. Biodistribution studies of the 24-h pretargeting strategy

S10. Pretargeted PET imaging of the MDA-MB-231 tumor at left shoulder

S11. Coronal and transverse 2D sectional PET images of conventional strategy in MDA-MB-231 tumor mice

S12. Coronal and transverse 2D sectional PET images of pretargeting strategy in MDA-MB-231 tumor mice

S13. Gall bladder coronal and transverse 2D sectional PET images of pretargeting strategy in MDA-MB-231 tumor mice

Table S1. Tumor-to-organ ratio by both conventional strategy and pretargeting strategy

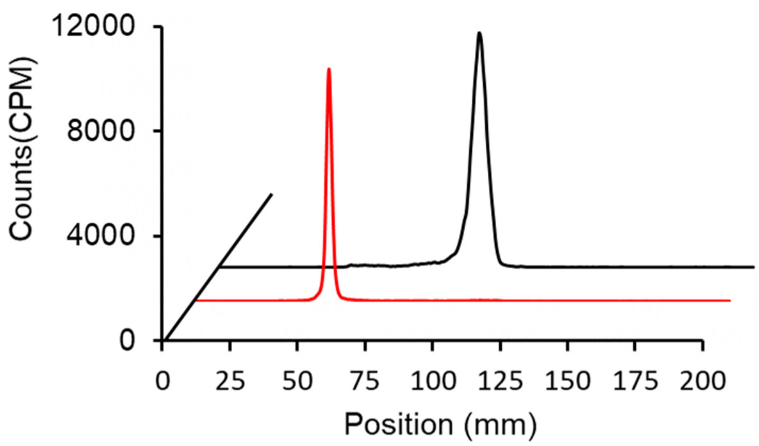

Figure S1. Radio-TLC of radioimmunoconjugate ^{64}Cu-PCB-TE2A-Tz clicked *ex vivo* with hnRNP-PEG$_4$-TCO (red) and only radio chelator (black) performed on iTLC-SG, 25% 0.1 M NH_4OAc:MeOH.

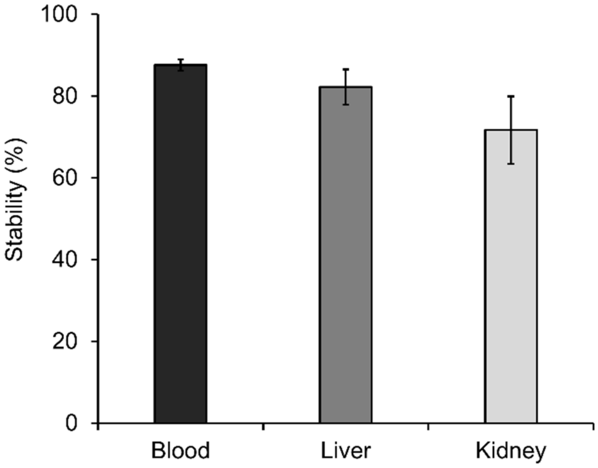

Figure S2. *In vivo* stability studies of the radioligand ^{64}Cu-PCB-TE2A-Tz in blood, liver, and kidneys of the BALB/c mice (n = 3).

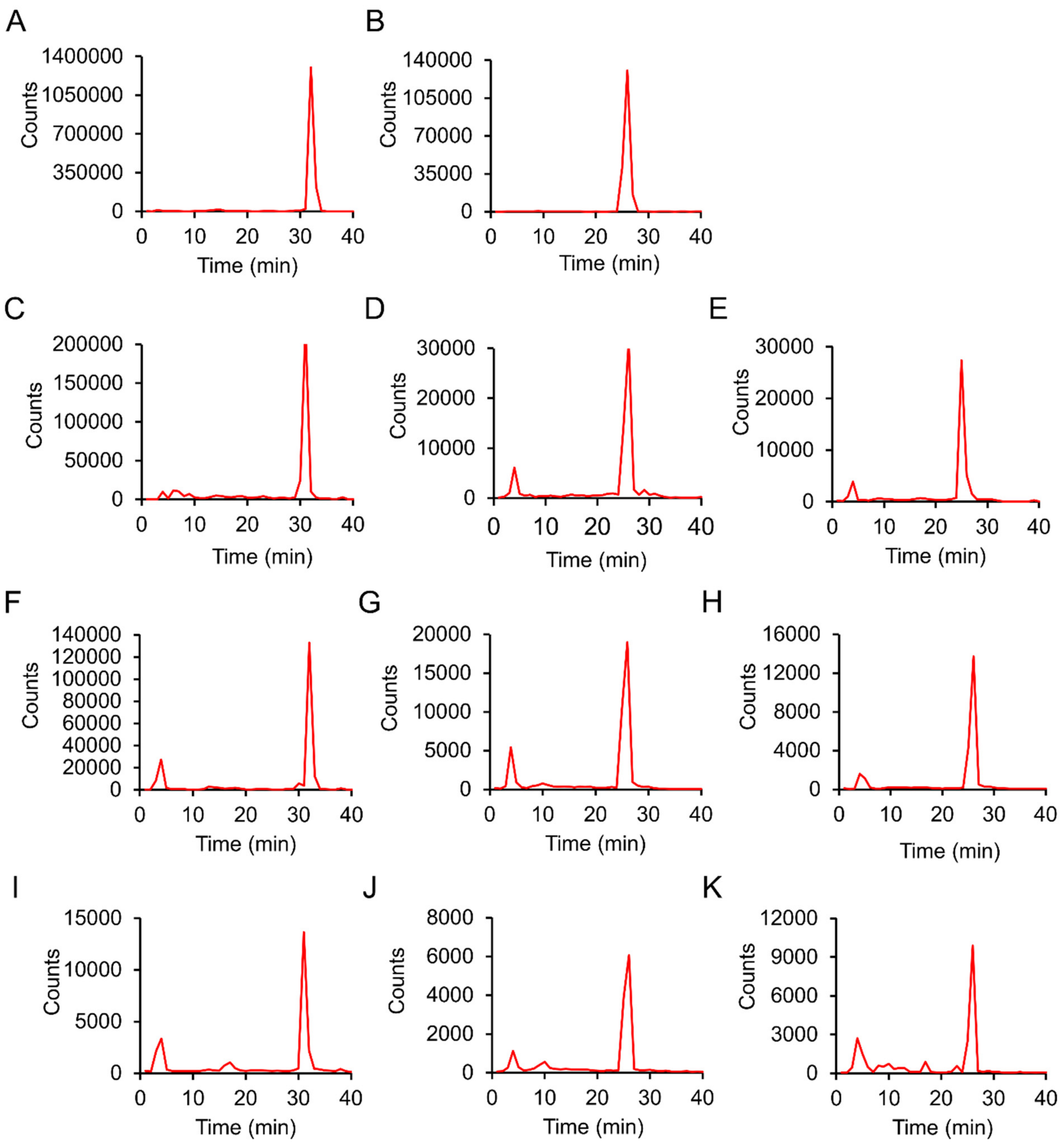

Figure S3. HPLC-chromatograms demonstrating *in vivo* stability of ^{64}Cu-PCB-TE2A-Tz (n = 3). Chromatograms of intact ^{64}Cu-PCB-TE2A-Tz in two different HPLC systems (**A, B**) blood metabolites (**C**-**E**), liver metabolites (**F**-**H**), and kidney metabolites (**I**-**K**). The peaks at 25 min and 30 min represent intact ^{64}Cu-PCB-TE2A-Tz radiochelator; peaks around 3 min represent free $^{64}Cu^{2+}$ ions and small peaks between free $^{64}Cu^{2+}$ ions and intact chelator demonstrate metabolized fractions.

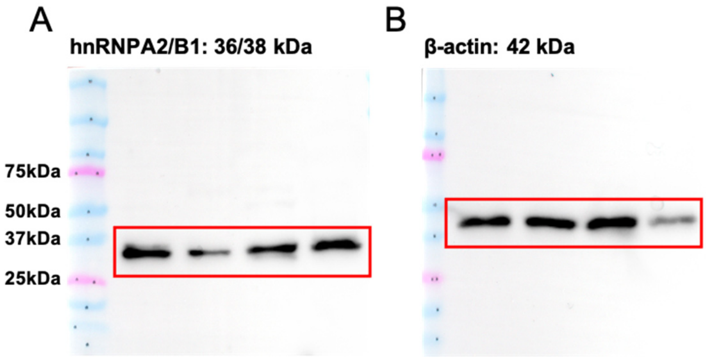

Figure S4. The uncropped version of western blot analysis of hnRNPA2B1 expression in various cells lines (**A**) hnRNPA2B1: ~36/38 kDa, (**B**) β-actin: ~42 kDa: Lane 1: MDA-MB-231, Lane 2: U87MG, Lane 3: MCF-7, Lane 4: HepG2.

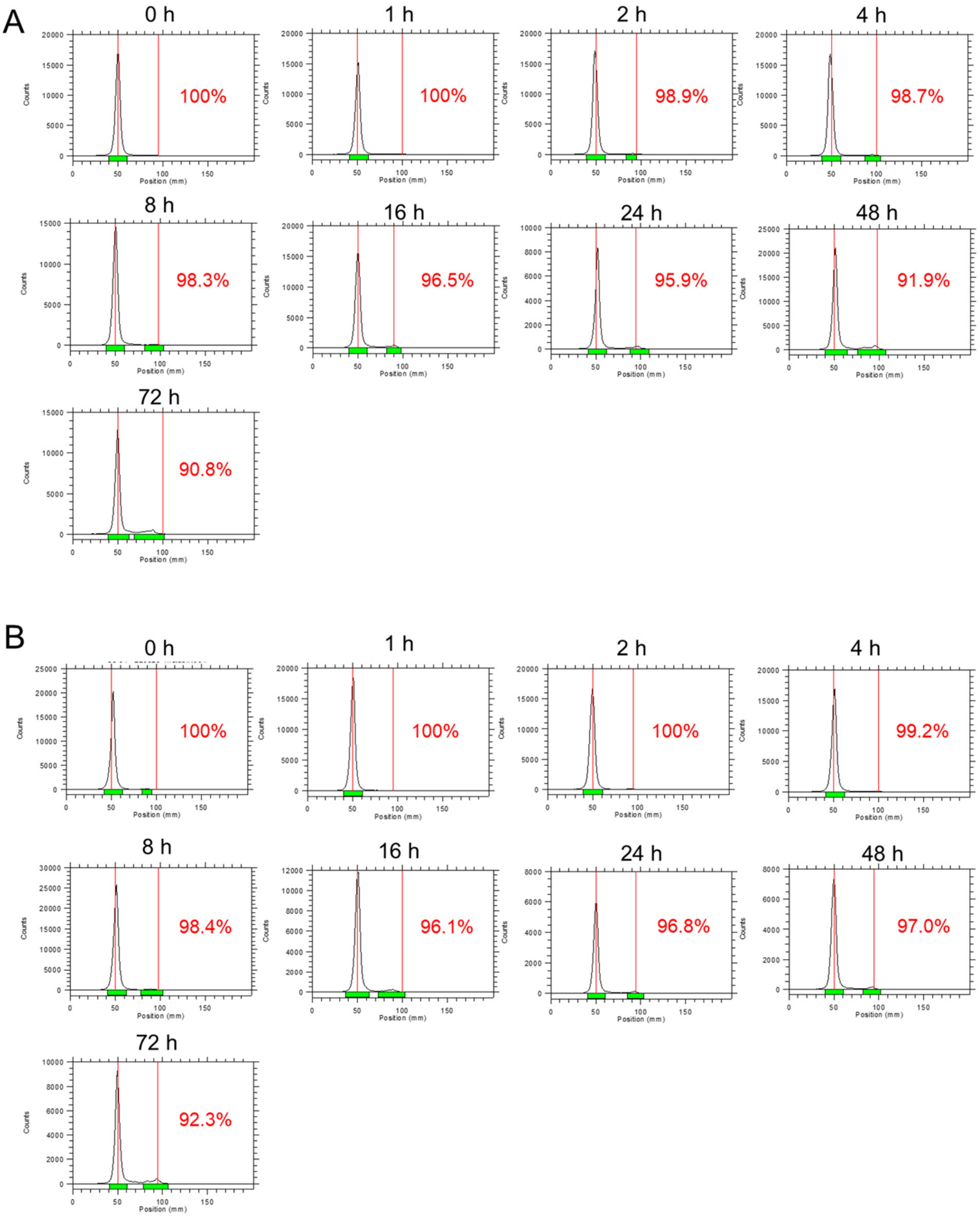

Figure S5. Representative radio-TLC analysis chromatograms of *in vitro* stability studies of the radioimmunoconjugate ^{64}Cu-PCB-HnRNP from triplicate experiment. ^{64}Cu-PCB-HnRNP was incubated in phosphate buffered saline (PBS) (**A**) and fetal bovine serum (FBS) (**B**) at 37 °C and spotted on iTLC-SG, to run in 25% 0.1 M NH_4OAc:MeOH at assigned time-points.

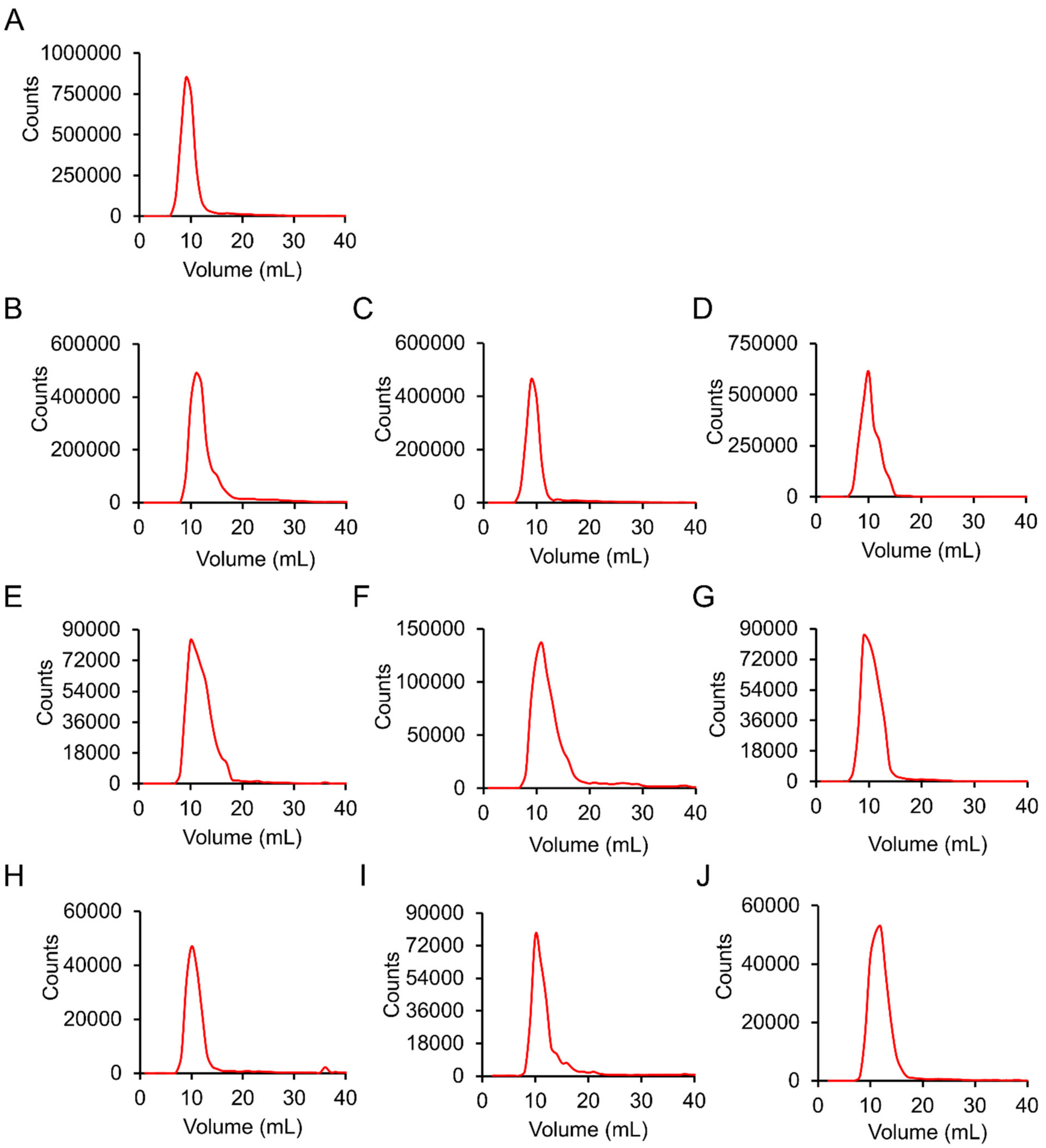

Figure S6. *In vivo* stability of the radioimmunoconjugate ^{64}Cu-PCB-HnRNP was assayed by PD-10 size exclusion columns. The stability studies were carried out triplicate in blood, liver, and kidneys of the BALB/c mice. SEC chromatograms for intact ^{64}Cu-PCB-HnRNP (**A**), blood sample (**B**-**D**), liver sample (**E**-**G**), and kidney sample (**H**-**J**) were shown.

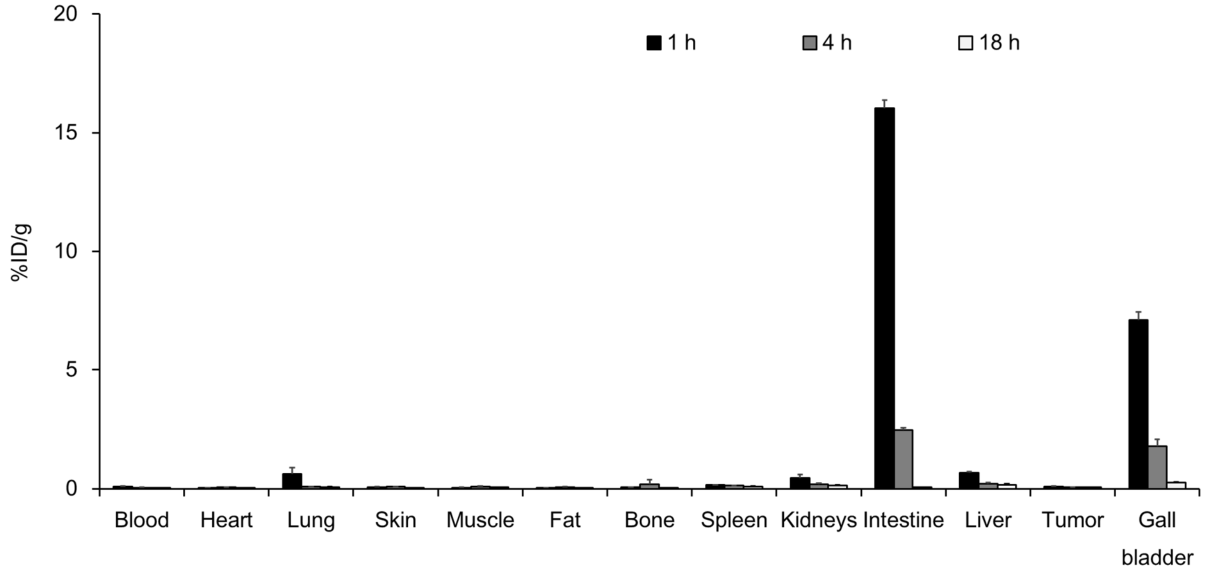

Figure S7. Biodistribution of radiolabeled ^{64}Cu-PCB-TE2A-Tz chelator in female nude mice incorporating MDA-MB-231 tumor xenograft.

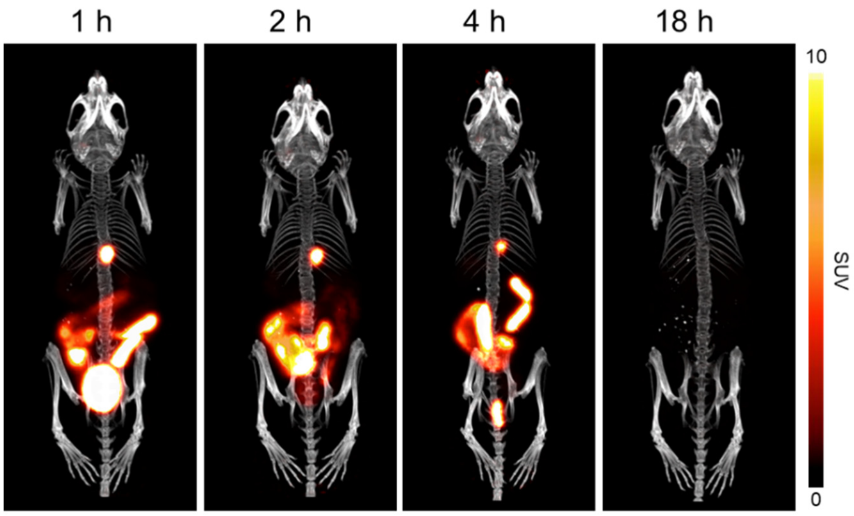

Figure S8. Clearance monitoring PET/CT images of the radiolabeled ^{64}Cu-PCB-TE2A-Tz chelator in female nude mice incorporating MDA-MB-231 tumor xenograft.

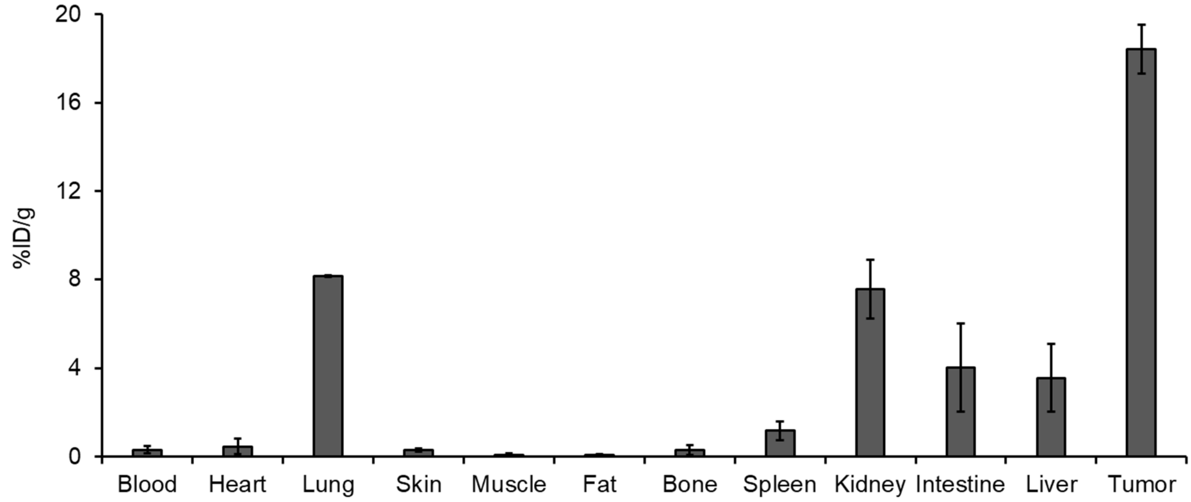

Figure S9. Biodistribution studies of pretargeting strategy (24-h) in the female nude mice bearing MDA-MB-231 tumor xenografts. Organs were harvested at 4-h time point after the administration of ^{64}Cu-PCB-TE2A-Tz.

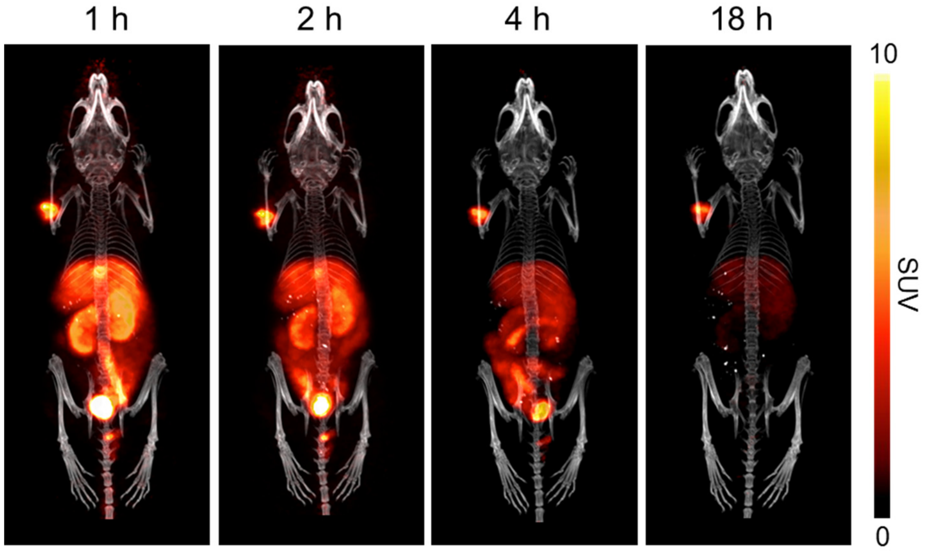

Figure S10. PET/CT images of pretargeting strategy of hnRNP-PEG4-TCO mAb 48-h prior to the injection of ^{64}Cu-PCB-TE2A-Tz in the female nude mice incorporating the MDA-MB-231 tumor xenograft at left shoulder.

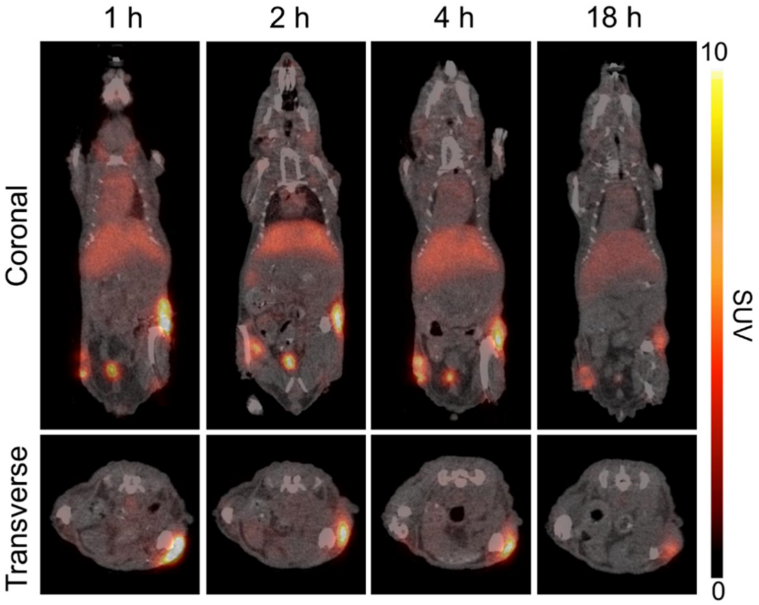

Figure S11. 2D sectional PET images of the conventional strategy with ^{64}Cu-PCB-hnRNP in female nude mice incorporating the MDA-MB-231 tumor xenograft at mammary fat pad (left-hand side) and flank (right-hand side) for coronal section and transverse section flank tumor.

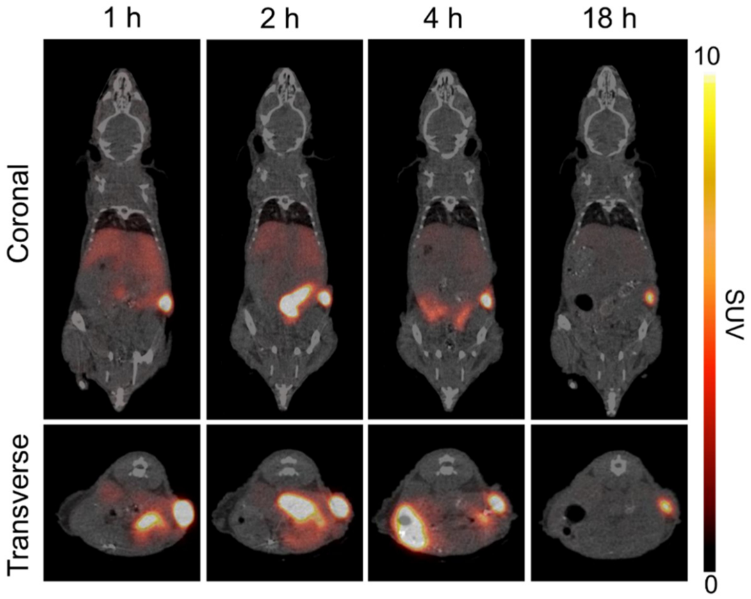

Figure S12. Coronal and transverse 2D sectional PET images of the pretargeting strategy by hnRNP-PEG4-TCO mAb 48-h prior injection of the ^{64}Cu-PCB-TE2A-Tz in the female nude mice incorporating MDA-MB-231 tumor xenograft.

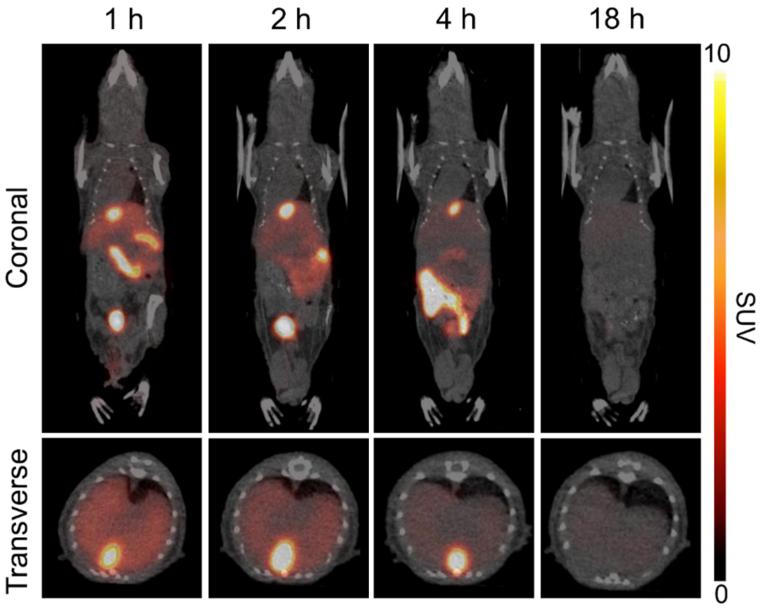

Figure S13. Gallbladder coronal and transverse 2D sectional PET images of pretargeting study by hnRNP-PEG$_4$-TCO mAb 48-h prior to the injection of ^{64}Cu-PCB-TE2A-Tz in the female nude mice incorporating the MDA-MB-231 tumor xenograft.

Table S1. Tumor-to-organ ratio of conventional strategy and pretargeting strategy.

**Organs**	**Conventional (Tumor 1)**		**Conventional (Tumor 2)**		**Pretargeting (24 h)**	**Pretargeting (48 h)**	
	4 h	18 h	4 h	18 h	4 h	4 h	8 h
Muscle	13.6	65.2	14.3	62.3	188.1	314.1	558.5
Blood	2.9	5.5	3.1	5.3	57	92.2	127.5
Liver	0.6	3.7	0.7	3.5	5.2	10.5	10.8
Intestine	9.0	21.9	9.4	20.9	4.5	6.4	4.7
Lung	9.0	16.9	9.4	16.1	2.2	4	7.2
Kidney	0.4	1.3	0.4	1.3	2.4	3.7	4.1
